# Evaluation of factors associated the expression of anti-HBs in children in Hunan Province, China

**DOI:** 10.1186/s12887-022-03718-z

**Published:** 2022-12-06

**Authors:** Shan Tan, Shizhou Li, Jianxiang Dong, Hongmei Dai, Minghua Yang, Fang Sun, Yang Yang, Jie Jiang, Shan Zhu, Mingyi Zhao, Zhiheng Chen

**Affiliations:** Department of Paediatrics, The Third Xiangya Hospital, Central South University, Changsha, Hunan People’s Republic of China

**Keywords:** HBV vaccine, Hepatitis B surface antibody, Vitamin D, Children

## Abstract

**Background and objectives:**

Vaccine is the most essential avenue to prevent hepatitis B virus (HBV) infection in infants and preschool children in China, with the largest populations carrying HBV in the world. This study aimed to evaluate the factors associating the response level of anti-HBs in children, providing instructions for HBV prevention clinically.

**Methods:**

The children taking physical examinations in the Third Xiangya Hospital from January 2013 to April 2020 were recruited. Telephone follow-up were adopted to collect further information. Univariate logistic regression was used to analyse the relationship between age and anti-HBs expression. Grouping by age and anti-HBs expression, we used chi-square test and T test to compare qualitative and quantitative data between positive group and negative group in each age subgroup. The meaningful variables (*P* < 0.10) in chi-square test or T test were further assessed with collinearity and chosen for univariate and multivariate logistic regression analysis by the stepwise backward maximum likelihood method (α_in_ = 0.05, α_out_ = 0.10).

**Results:**

A total of 5838 samples (3362 males, 57.6%) were enrolled. In total, the incidence of negative anti-HBs increased with age[OR = 1.037(1.022–1.051)]. Multivariate logistic regression analysis illustrated that anemia[OR = 0.392(0.185–0.835)], age[OR = 2.542(1.961–3.295)] and Vit D[OR = 0.977(0.969–0.984)] in 0.5–2.99 years subgroup, Zinc deficiency[OR = 0.713(0.551–0.923] and age[OR = 1.151(1.028–1.289)] in 3–5.99 years subgroup, Vit D[OR = 0.983(0.971–0.995)] in 12–18 years subgroup had significant association with anti-HBs.

**Conclusions:**

This retrospective study illustrated that age, anemia status, zinc deficiency and vitamin D were associated with anti-HBs expression in specific age groups of children, which could serve as a reference for the prevention of HBV.

**Supplementary Information:**

The online version contains supplementary material available at 10.1186/s12887-022-03718-z.

## Background

Hepatitis B virus infection has been a worldwide sanitary problem threatening individuals’ health quality. According to a report of World Health Organization, in 2015, the global prevalence of HBV infection in the general population was estimated at 3.5%, nearly 257 million people suffered from chronic HBV infection worldwide and 887,220 persons died of HBV infection [1]. China had the largest citizens carrying HBV. In a study conducted during 2013–2017, the prevalence of HBV infection in the general population of China from 2013 to 2017 was 6.89% (95% CI: 5.84–7.95%), and predicted there were 83,864,139 (95% CI: 60,406,793-110,751,614) infected case in 2018 [2].

HBV transmission happening in the perinatal period and early childhood is an important pattern of transmission [3]. To prevent HBV infection in infants and preschool children and control HBV infection in China, it’s essential to stimulate the hepatitis B vaccination of children. In 2002, the Chinese authority implemented a new vaccine strategy in which vaccines listed in China’s National Immunization Programme, including the hepatitis B vaccine, were offered freely to neonates and infants. All full-term newborns were given hepatitis B vaccine at 0–1-6 month schedule after birth to block transmission from mother to fetus [4, 5], which was consistent with the vaccination schedule recommended by the United States and other developed countries [6]. After this, the hepatitis B vaccine coverage rate increased significantly as the HBsAg prevalence also decreased in China. In a meta-analysis from 2013 to 2017, HBV prevalence in children younger than 15 years old was lower than 2.1% as a result of the HBV immunization scheme for infants [2]. Not coincidentally, an epidemiological survey showed that HBsAg prevalence declined from 10.5 to 0.8% among children < 15 years and 9.9 to 0.3% among children < 5 years during 1992–2014 [7].

Several studies have evaluated the tangible ability to maintain the level of the anti-HBs of the classical pattern of HBV immunization where individuals are injected with the vaccine at 0, 1, and 6 months after birth. First, the time to completion of vaccination is the primary factor. Xiaomei Yue followed the serum anti-HBs levels of Chinese children who received the hepatitis B vaccine as planned. The positive rates of serum anti-HBs(> = 10mIU/ml) were 16,190/17928(90.31%) for 1 year, 10,755/12808(83.95%) for age 2, 6855/9543(71.82%) for age 3, 4997/8042(62.14%) for age 4 [8]. In another study, positive rates of serum anti-HBs for 1–4 years old Chinese children were 79/83(95.3%) for 1 year, 47/53(88.2%) age 2, 44/61(72.1%) for age 3, 82/141(58.2%) for age 4 [9]. The results of the above two studies showed that the positive rate of anti-HBs decreased rapidly during the period from 1 to 3 years old and gradually stabilized after about 4 years old. In addition, the status of maternal immunization [10], large for gestational age [11], dosing schedule [12], vaccinate dosage [13] and mother’s educational background [14] were also reported. However, there is still an immense absence of objective factors influencing the response level associated with the individuals themselves, such as their nutrition condition and the method of birth.

Studies in adults have tested other variables that may affect immune status among those receiving vaccines. Variables have been determined to decrease response levels include the site of injection (the gluteal site of injection decreases its efficiency) [15], obesity [16, 17]^,^ cigarette smoking, older age [15–17] and the presence of diseases that alter the immune system [17]. No previous studies have conducted a systematic review targeting these variables in children.

In this paper, we evaluate the influence of multiple factors ranging from the detailed objective biochemistry index value to different avenues of birth and feeding on the immunization level of hepatitis B vaccine among children. We also sought to determine the potential co-relationship between different factors affecting that relationship, and provide instruction for hepatitis B prevention clinically.

## Methods

### Participant recruitment

Children between 6 months and 18 years old attending physical examinations at the Third Xiangya Hospital of Central South University, Changsha, Hunan, between January 2013 and April 2020, were recruited in this study (*n* = 100,997). Firstly, we eliminated participants who did not undergo anti-HBs detections (*n* = 56,181) or were detected HBsAg positive (*n* = 57). Thereafter, we established precise inclusion criteria for the remaining candidates having negative HBsAg (*n* = 44,759) to generate valid subjects. Herein, participants lacking essential information such as Gender, Age, way of birth, BMI (*n* = 9180) were not eligible. After which, individuals who were low birth weight (< 2500 g, *n* = 242), premature (< 37 weeks, *n* = 430), combined with diseases which might affect the response level of immunization, such as organic disease related to heart, lung, liver and kidney, allergic disease including asthma, anaphylactoid purpura and other immunological diseases (*n* = 556) were excluded. Next, we filtered subjects failing to follow the obligational hepatitis B vaccination plan, where children would be vaccinated within 24 hours, 1 month, and 6 months (*n* = 10,887) as well as having revaccination before this physical examination (*n* = 15,150). Then we obtained further information from mentioned participants (*n* = 8314), including duration of the lactation period, history of eczema, frequency of cold and exposure to second-hand cigarette smoke by telephone follow-up. Herein, subjects who were unable to provide precise and complete data ascribed to memory bias or loss to follow-up (*n* = 1206) were eliminated. Then we excluded participants whose mothers had ever been infected with hepatitis B before delivery or given hepatitis B immune globulin at birth (*n* = 1270). The final candidates (*n* = 5838) were divided into anti-HBs-positive group (*n* = 3824) and anti-HBs-negative group (*n* = 2014). An additional flow chart shown research design in more detail (see Additional file 1). This research was approved by the ethics committee of the Third Xiangya Hospital of Central South University and followed medical ethics. Parental consent in person was achieved for participants under 18 years old.

### Statistics reference

The references for statistics were illustrated in Additional file 2 and Additional file 3, including status of anemia, body mass index, level of microelements and the classification standard of anti-HBs. For children younger than 6 years old, growth curve or weight of height is used to evaluate nutrition rather than BMI, whose classification process is cumbersome, so this study will not discuss it. We applied the standard of inhalation of environmental tobacco smoke as the definition of being exposed to SHS [18] and more than 6 colds per year as a definition of frequent colds.

### Statistical analysis

Enumeration data were described by n (%). Measurement data meeting the criterion of normal distribution were shown by means and standard deviations. All variables were processed by univariate logistic regression analysis and odds ratios (ORs) and 95% confidence intervals (CIs) were utilized to assess the extent of the relevance. The variables which *P* value < 0.10 in the chi-square test and T test in any age subgroup were selected for univariate logistic regression analysis, then the variables which P value < 0.10 were selected for further multivariate logistic regression analysis for each age subgroup separately. Scatter plots and fitting lines (loess method, a t-based approximation) were drawn using R language (version 3.6.3) “ggplot2” package. Data were mostly analysed through SPSS (IBM SPSS Statistics for Macintosh, Version 24.0, Armonk, NY: IBM Corp). *P* < 0.05 was considered statistically significant.

## Results

A total of 5838 children were enrolled in this study, including 2476/5838 (42.4%) girls and 3362/5838 (57.6%) boys, among whom there were 2014/5838 (34.5%) anti-HBs-negative children and 3824/5838 (65.5%) anti-HBs-positive children. As mentioned earlier, time to vaccination has a significant impact on vaccine response rates. Therefore, we adopt the univariable logistics regression to analyse the relationship between age and anti-HBs expression. In the whole samples, for each additional year of age, the probability of anti-HBs negative increased by 3.7%[OR = 1.037(1.022–1.051)]. Since age has a great influence on anti-HBs expression, we divided the samples into four subgroups according to the characteristics of children’s development. The 0.5–2.99 years subgroup has 1166 samples, among them, 913/1166(78.3%) samples were anti-HBs positive. The 3–5.99 years subgroup has 1680 samples, among them, 1025/1680(61%) samples were anti-HBs positive. The 6–11.99 years subgroup has 2265 samples, among them, 1407/2265(61.8%) samples were anti-HBs positive. The 12–18 years subgroup has 727 samples, among them, 479/727(65.9%) samples were anti-HBs positive.

Each age subgroup was further divided into the anti-HBs positive group and negative group. The Chi-square test was applied to analyse enumeration data and T test was applied to analyse measurement data conforming to normal distribution between the anti-HBs positive group and negative group. As the result shown in Tables 1 and 2, in the 0.5–2.99 years subgroup, anemia(*P* < 0.001), age(*P* < 0.001), WBC(*P* = 0.011) and Vit D(*P* < 0.001); in the 3–5.99 years subgroup, zinc deficiency(*P* = 0.022) and age(*P* = 0.014); in the 12–18 years subgroup, Vit D(*P* = 0.012) have significant difference between anti-HBs positive and anti-HBs negative group.

**Table 1:** Chi-square test results between antibody positive and antibody negative group in four age subgroups

Variable	0.5 ~ 2.99 years(*n* = 1166)			3 ~ 5.99 years(*n* = 1680)			6 ~ 11.99 years(*n* = 2265)			12 ~ 18 years(*n* = 727)		
	AntiHBs Expression		*P*	AntiHBs Expression		*P*	AntiHBs Expression		*P*	AntiHBs Expression		*P*
	Positive (*n* = 913)	Negative (*n* = 253)		Positive (*n* = 1025)	Negative (*n* = 655)		Positive (*n* = 1407)	Negative (*n* = 828)		Positive (*n* = 479)	Negative (*n* = 248)	
**Gender**												
Female	403	119	0.412	432	279	0.856	609	380	0.640	173	81	0.354
Male	510	134		593	376		798	478		308	167	
**Birth way**												
eutocia	422	115	0.829	520	341	0.595	764	453	0.487	247	137	0.347
cesarean section	491	138		505	314		643	405		232	111	
**BMI**	–	–	–	–	–	–						
thin	–	–	–	–	–	–	116	66	0.131	54	41	0.133
normal	–	–	–	–	–	–	1040	665		336	162	
overweight or obese	–	–	–	–	–	–	251	127		89	45	
**Duration of lactation period (month),**												
0~	285	82	0.150	328	208	0.875	439	257	0.311	160	86	0.557
6~	455	111		503	319		692	449		224	121	
12~	173	60		190	128		276	152		95	41	
**Whether the children had a history of eczema**												
Yes	318	93	0.570	327	225	0.297	443	220	0.253	143	82	0.375
No	595	160		698	430		964	568		336	166	
**Whether the children had a history of second-hand cigarette exposure**												
Yes	259	66	0.474	284	195	0.361	431	233	0.078*	142	72	0.864
No	654	187		741	460		976	625		337	176	
**Whether the children had a frequent cold**												
Yes	214	67	0.317	258	168	0.828	349	203	0.538	114	58	0.901
No	699	186		767	487		1058	655		365	190	
**Anemia**												
Yes	97	8	< 0.001***	85	42	0.155	112	58	0.293	20	10	0.927
No	816	245		940	613		1295	800		459	238	
**Lead intoxication**												
Yes	3	0	0.832	2	2	0.999	8	1	0.097*	4	2	0.968
No	910	253		1023	653		1399	857		475	248	
**Cadmium intoxication**												
Yes	29	14	0.078*	17	12	0.790	35	26	0.439	11	7	0.665
No	884	239		1008	643		1372	832		468	241	
**Zinc deficiency**												
Yes	673	174	0.119	159	130	0.022**	988	632	0.079*	185	112	0.089*
No	240	79		866	525		419	226		294	136	
**Copper deficiency**												
Yes	118	29	0.535	214	161	0.076*	412	264	0.453	177	100	0.375
No	796	224		811	494		995	594		302	148	
**Calcium deficiency**												
Yes	4	2	0.844	35	27	0.453	436	258	0.646	326	153	0.086*
No	909	251		990	628		971	600		153	95	
**Iron deficiency**												
Yes	490	118	0.095*	388	236	0.451	286	176	0.915	28	16	0.745
No	433	135		637	419		1121	682		451	232	

*P < 0.10; **P < 0.05; ***P < 0.01

**Table 2:** T test results between antibody positive and antibody negative group in four age subgroups

Variable	0.5 ~ 2.99 years(*n* = 1166)			3 ~ 5.99 years(*n* = 1680)			6 ~ 11.99 years(*n* = 2265)			12 ~ 18 years(*n* = 727)		
	Anti-HBs Expression ($\overline{\textrm{X}}$ ±SD)		*P*	Anti-HBs Expression ($\overline{\textrm{X}}$ ±SD)		*P*	Anti-HBs Expression ($\overline{\textrm{X}}$ ±SD)		*P*	Anti-HBs Expression ($\overline{\textrm{X}}$ ±SD)		*P*
	Positive (*n* = 913)	Negative (*n* = 253)		Positive (*n* = 1025)	Negative (*n* = 655)		Positive (*n* = 1407)	Negative(*n* = 828)		Positive (*n* = 479)	Negative(*n* = 248)	
**Age**	1.790 ± 0.663	2.129 ± 0.506	< 0.001***	4.291 ± 0.867	4.398 ± 0.879	0.014**	8.781 ± 1.668	8.697 ± 1.700	0.247	13.657 ± 1.226	13.608 ± 1.224	0.610
**WBC(10^9/L)**	8.596 ± 2.395	8.236 ± 1.857	0.011**	7.659 ± 2.109	7.571 ± 2.064	0.405	6.896 ± 1.950	6.870 ± 1.801	0.746	6.469 ± 1.560	6.487 ± 1.528	0.882
**VitD(ng/ml)**	48.343 ± 18.992	40.810 ± 17.443	< 0.001***	45.071 ± 15.604	43.853 ± 14.831	0.112	41.966 ± 13.144	41.354 ± 13.413	0.286	39.209 ± 11.843	36.751 ± 12.778	0.012**
**C3(g/L)**	1.027 ± 0.198	1.030 ± 0.196	0.814	1.025 ± 0.220	1.021 ± 0.183	0.670	1.021 ± 0.203	1.024 ± 0.186	0.758	1.031 ± 0.183	1.032 ± 0.175	0.954
**C4(g/L)**	0.243 ± 0.144	0.255 ± 0.142	0.224	0.252 ± 0.141	0.246 ± 0.138	0.324	0.248 ± 0.137	0.252 ± 0.137	0.469	0.245 ± 0.139	0.241 ± 0.134	0.695
**IgA(g/L)**	1.433 ± 0.720	1.408 ± 0.722	0.619	1.405 ± 0.739	1.391 ± 0.700	0.709	1.462 ± 0.719	1.443 ± 0.724	0.555	1.557 ± 0.750	1.577 ± 0.707	0.732
**IgG(g/L)**	10.443 ± 2.237	10.436 ± 2.271	0.967	10.304 ± 2.290	10.165 ± 2.416	0.235	10.393 ± 2.277	10.417 ± 2.234	0.808	10.452 ± 2.207	10.650 ± 2.264	0.257
**IgE(IU/mL)**	149.414 ± 86.725	148.166 ± 87.992	0.840	155.292 ± 130.971	147.866 ± 102.403	0.219	151.545 ± 134.349	150.814 ± 158.919	0.907	156.317 ± 139.988	155.264 ± 131.955	0.992
**IgM(g/L)**	1.25 ± 0.71	1.31 ± 0.75	0.209	1.265 ± 0.709	1.241 ± 0.722	0.518	1.251 ± 0.725	1.261 ± 0.734	0.743	1.218 ± 0.719	1.175 ± 0.700	0.442

*P < 0.10; **P < 0.05; ***P < 0.01

**Fig. 1:**
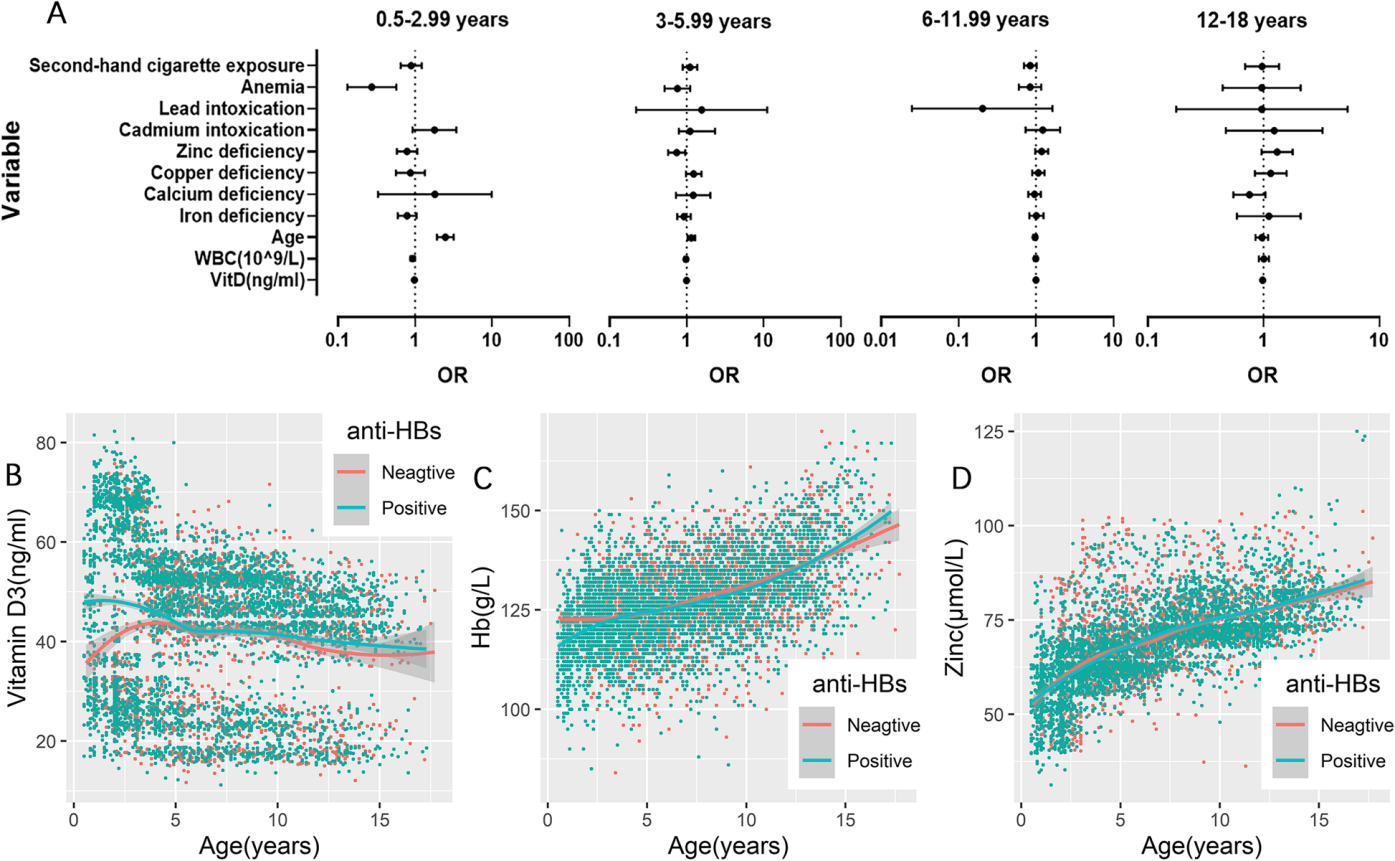
**A** Forest map of univariable logistics regression analysis. **B-D** Scatter plots and fitting lines showed the distribution relationship between age and Vit D, hemoglobin(Hb) and Zinc. The red dots represented the anti-HBS negative samples, while green dots represented the positive. Fitting line were drawn by loess method, whose grey shadow indicated 95% confidence interval

Subsequently, variables with *P* < 0.1 in chi-square and T test were included in the univariable logistics regression analysis. In the 0.5–2.99 years subgroup, anemia [OR = 0.275(0.132–0.573), *P* < 0.001], WBC[OR = 0.93(0.872–0.992), *P* = 0.028] and VitD [OR = 0.978(0.971–0.986), *P* < 0.001] were protective factor for anti-HBs expression, while age [OR = 2.476(1.932–3.172), *P* < 0.001] was risk factor. The probability of anti-HBs negative increased by 147.6% for each age. In the 3–5.99 years subgroup, sufficient zinc [OR = 0.713(0.551–0.923, *P* = 0.01)] was protective factor for anti-HBs expression, while age [OR = 1.151(1.028–1.289), *P* = 0.014] was risk factor. In the 6–11.99 years subgroup, no statistically significant influencing factors were obtained. In the 12–18 years subgroup, VitD [OR = 0.984(0.972–0.996), *P* = 0.01] was protect factor for anti-HBs expression (Table 3, Fig. 1A). The OR of age, WBC and VitD means each additional unit of these variables increases the probability of anti-HBs negative for (OR-1) × 100%. To further evaluate the distribution relationship between age and other measurement data, as well as the influence on anti-HBS expression, scatter plots and fitting lines were drawn using R language. Mean VitD levels were significantly higher in the anti-HBS positive group than negative group when age < 4 years (Fig. 1B). When it came to Hb, anti-HBs negative children under 2 years had higher hemoglobin level (Fig. 1C). There was no significant difference in the quantitative level of zinc among different age groups, as zinc was discussed as a categorical variable in univariate logistics analysis (Fig. 1D).

**Table 3:** Univariable logistic regression analyze results of four age subgroups

Variable	0.5 ~ 2.99 years(*n* = 1166)		3 ~ 5.99 years(*n* = 1680)		6 ~ 11.99 years(*n* = 2265)		12 ~ 18 years(*n* = 727)	
	OR(95%CI)	*P* value	OR(95%CI)	*P* value	OR(95%CI)	*P* value	OR(95%CI)	*P* value
**Whether the children had a history of second-hand cigarette exposure**								
No	0.891(0.650–1.221)	0.474	1.106(0.891–1.347)	0.361	0.844(0.699–1.019)	0.078*	0.971(0.693–1.361)	0.864
Yes^a^								
**Anemia**								
No	0.275(0.132–0.573)	< 0.001***	0.758(0.516–1.112)	0.156	0.838(0.603–1.166)	0.293	0.964(0.444–2.092)	0.927
Yes^a^								
**Lead intoxication**								
No			1.567(0.22–11.111)	0.645	0.204(0.025–1.634)	0.134	0.965(0.176–5.319)	0.968
Yes^a^								
**Cadmium intoxication**								
No	1.786(0.929–3.436)	0.082*	1.106(0.525–2.331)	0.79	1.225(0.732–2.409)	0.44	1.236(0.473–3.226)	0.666
Yes^a^								
**Zinc deficiency**								
No	0.786(0.580–1.065)	0.119	0.741(0.574–0.958)	0.022**	1.186(0.980–1.435)	0.079*	1.309(0.96–1.786)	0.089*
Yes^a^								
**Copper deficiency**								
No	0.873.(0.566–1.344)	0.536	1.235(0.978–1.56)	0.076*	1.073(0.892–1.292)	0.453	1.153(0.842–1.580)	0.375
Yes^a^								
**Calcium deficiency**								
No	1.812(0.330–9.901)	0.494	1.217(0.729–2.028)	0.454	0.958(0.796–1.152)	0.646	0.756(0.549–1.041)	0.087*
Yes^a^								
**Iron deficiency**								
No	0.789(0.596–1.043)	0.095*	0.925(0.755–1.134)	0.451	1.011(0.82–1.248)	0.915	1.111(0.589–2.096)	0.745
Yes^a^								
**Age**	2.476(1.932–3.172)	< 0.001***	1.151(1.028–1.288)	0.014**	0.971(0.923–1.021)	0.247	0.968(0.853–1.098)	0.609
**WBC(10^9/L)**	0.93(0.872–0.992)	0.028**	0.98(0.935–1.028)	0.405	0.993(0.949–1.038)	0.746	1.008(0.913–1.112)	0.882
**VitD(ng/ml)**	0.978(0.971–0.986)	< 0.001***	0.995(0.988–1.001)	0.112	0.997(0.99–1.003)	0.286	0.984(0.972–0.996)	0.010**

^a^Reference group; *P < 0.10; **P < 0.05; ***P < 0.01

Then, variables with *P* < 0.1 in the univariable logistics regression analysis were included into multivariable logistics regression. Stepwise backward maximum likelihood method was applied to eliminate meaningless variables (α_in_ = 0.05, α_out_ = 0.10). Compare to univariate logistic, the WBC was excluded in multivariable logistics regression (Table 4).

**Table 4:** Multivariable logistics regression analyze results of four age subgroups

Variable	OR(95%CI)	*P* value
**0.5 ~ 2.99 years(*n*** **= 1166)**		
**Anemia**		
No	0.392(0.185–0.835)	0.015*
Yes^a^		
**Cadmium intoxication**		
No	1.88(0.93–3.802)	0.079
Yes^a^		
**Age**	2.542(1.961–3.295)	< 0.001***
**VitD(ng/ml)**	0.977(0.969–0.984)	< 0.001***
**3 ~ 5.99 years(*n*** **= 1680)**		
**Zinc deficiency**		
No	0.713(0.551–0.923)	0.01*
Yes^a^		
**Copper deficiency**		
No	1.264(0.998–1.6)	0.052
Yes^a^		
**Age**	1.151(1.028–1.289)	0.015*
**6 ~ 11.99 years(*n*** **= 2265)**		
**Whether the children had a history of second-hand cigarette exposure**		
No	0.841(0.697–1.015)	0.072
Yes^a^		
**Zinc deficiency**		
No	0.84(0.695–1.016)	0.073
Yes^a^		
**12 ~ 17.99 years(*n*** **= 727)**		
**Zinc deficiency**		
No	1.355(0.991–1.855)	0.057
Yes^a^		
**VitD(ng/ml)**	0.983(0.971–0.995)	0.007**

^a^Reference group; *P < 0.05; **P < 0.01; ***P < 0.001

## Discussion

This research studied potential factors that may affect the response level of hepatitis B immunization, ranging from exterior elements such as the way of birth and duration of the lactation period to biochemical factors in the children. Our study demonstrated that age or time since vaccination remains a major factor associated with anti-HBs expression, which was consistent with previous studies [8, 9]. In the 0.5–2.99 years subgroup and the 3–5.99 years subgroup, the risk of negative anti-HBS was significantly increased with age, while the 6–11.99 years subgroup and the 12–18 years subgroup didn’t.

In this report, we found anemia acted as a protective factor statistically for the anti-HBs in the 0.5–2.99 years subgroup, especially < 2 years. However, there are still controversial opinions about the immune response in anemia patients. An American study showed that the prevalence of iron deficiency (ID), anemia, and iron deficiency anemia (IDA) among children 1–2 years (12–35.9 months) was 13.5% (9.8, 17.2), 5.4% (3.5, 7.4), and 2.7% (1.2, 4.2) respectively [19], which demonstrated anemia was a common child health problem. Continued breastfeeding may increase IDA prevalence in 6-to-23 months children, and the associations of anemia with inflammation, zinc deficiency and infections could be suggesting the occurrence of nutritional immunity [20]. A study of Kenyan infants clarified that correction of iron deficiency may improve the response to diphtheria, pertussis, and pneumococcal vaccines during early infancy [21]. Further research is needed on the mechanism and effect of anemia on the response to hepatitis B vaccination.

In our study, vitamin D was a significantly protective factor for anti-HBs in the 0.5–2.99 years subgroup and the 12–18 years subgroup by quantitative logistics regression analysis. These two ages subgroup correspond to the two main growth peaks for children -- infancy and adolescence. In the scatter plot, the effect of vitamin D deficiency was most obvious in children < 4 years. Vit D might have a beneficial impact on the body’s immune system and modulate both innate and adaptive immunity [22], which is also supported by our study. A clinical study illustrated that serum concentrations of 25(OH)D affect the level of IL-6, TNF-α, and CRP. Children with status ≥75 nmol/L had a high level of IL-6, TNF-α, and CRP compared to the < 50 nmol/L and 50–74.9 nmol/L categories [23]. Based on above results, 75 nmol/L is recommended as an appropriate vitamin D serum concentration.

Zinc is essential for cellular processes in all cells, especial for cells with a rapid rate of turnovers such as those of the immune, gastrointestinal systems, and skin. These cells are particularly vulnerable to zinc deficiency [24]. Interestingly, this effect was statistically significant only in the 3–5.99 subgroup in our research. The effect of zinc on vaccination remains to be further studied.

In contrast with studies aimed at adults [15], no association was confirmed between immune response and second-hand cigarette exposure. In fact, the detrimental outcome of cigarettes on immunologic reaction demands long-term exposure to smoke. Children usually expose to less smoke for a shorter time, making the adverse effect of smoking more formidable to discover.

This study was a retrospective, cross-sectional, case-control study. There was a certain lag before the observation of the outcome (the point of negative anti-HBs appearance). Older children may turn anti-HBs negative prior to the test. Therefore, we cannot determine the exact time when their antibodies turned negative. Cohort studies were needed to further investigate the long-term effects of early nutritional deficiencies. At the beginning of the research, anti-HBS quantitative titration experiment was not included in the routine item of physical examination, so we didn’t acquire complete quantitative anti-HBS data in this study. As a result, we only discussed qualitative anti-HBS level. Furthermore, our study did not distinguish anti-HBs positive groups(> = 10mIU/ml) and anti-HBs high response group(> = 100Miu/ml). In addition, the subjects of the research were selected from the children participating in voluntary physical examinations in Hunan Province, leading to inevitable selective bias. Although explicit inclusion criteria were applied, there was still avoidless memory bias of subtle details when completing the telephone follow-up survey.

## Conclusion

This study listed possible factors that might influence the response level to hepatitis B vaccines, providing a reference for both parents and health policy-makers. In the domain of efficient prevention of hepatitis, we recommended that parents should provide their children with healthy and balanced nutrition and sufficient Vit D, Zinc to maintain the quantity of antibodies, especially in the early neonatal period. In addition, enhancing the education of HBV infected mothers is a great way to block transmission of hepatitis B in children. Booster vaccination for children in high epidemic areas with anti-HBS turning negative is also a possible approach. Although this study found that anemia acted as a protective factor for anti-HBs in the 0.5–2.99 subgroup, there was no clear evidence that it was beneficial for the long-term maintenance of anti-HBs. We recommend that children with anemia should receive appropriate treatment as soon as possible to recover from anemia.

## Supplementary Information

**Additional file 1.** Participant flow chart.

**Additional file 2: Table S1.** The statistic reference of indicators.

**Additional file 3: Table S2.** The statistic reference of BMI.

## Data Availability

In consideration of the participants’ privacy right, the datasets used and/or analysed during the current study are available from the co-first author/corresponding author on reasonable request.

## Abbreviations

HBV: hepatitis B virus
GMT: geometric mean titer
BMI: body mass index
