# Supplementary figures and images for: Evaluation of factors associated the expression of anti-HBs in children in Hunan Province, China
Source: BMC Pediatr. 2022 Dec 6;22:697. doi: 10.1186/s12887-022-03718-z (PMC9724425; doi:10.1186/s12887-022-03718-z)

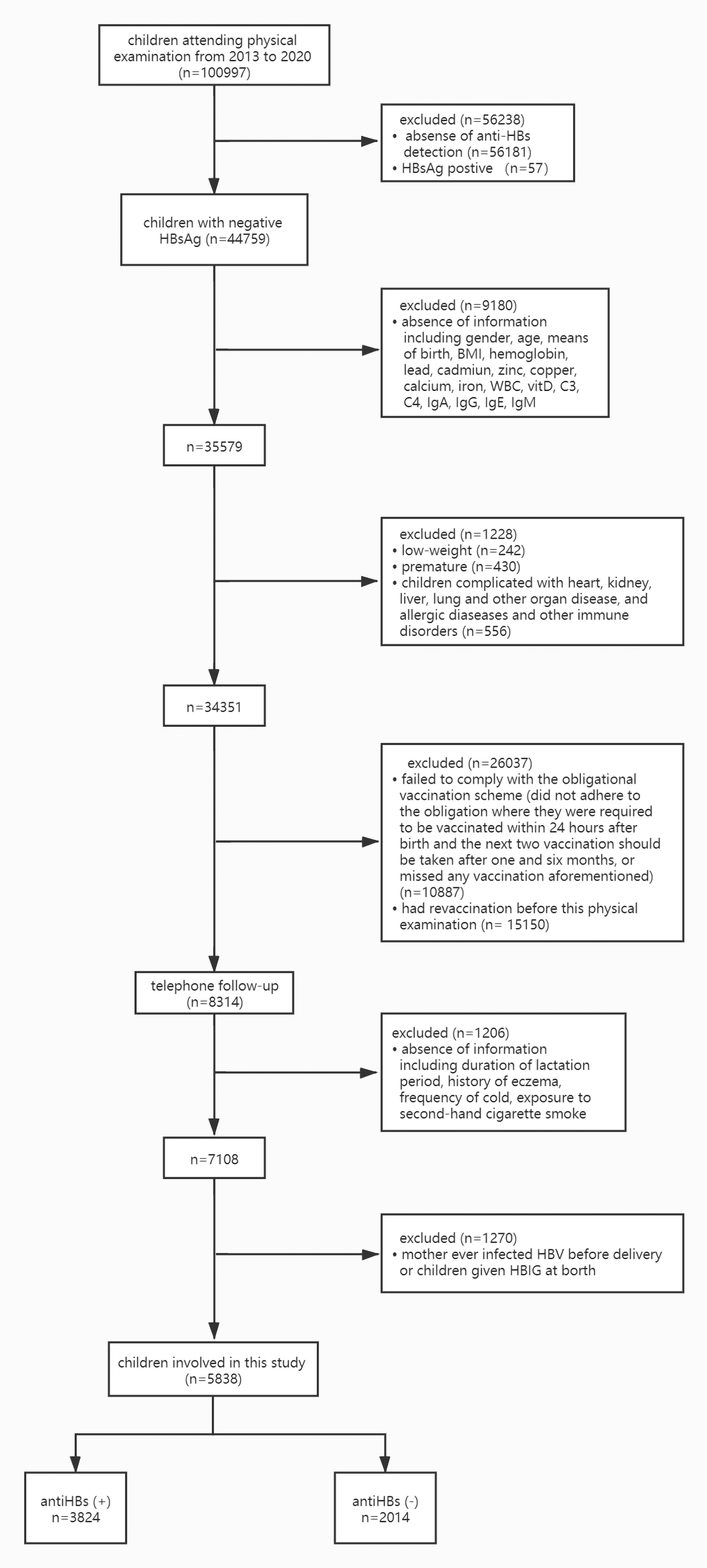

Supplement: Supplementary file 1 — Additional file 1. Participant flow chart. [file 12887_2022_3718_MOESM1_ESM.tiff]
