# Supplementary material for: Evaluation of factors associated the expression of anti-HBs in children in Hunan Province, China
Source: BMC Pediatr. 2022 Dec 6;22:697. doi: 10.1186/s12887-022-03718-z (PMC9724425; doi:10.1186/s12887-022-03718-z)
Supplement: Supplementary file 2 — Additional file 2: Table S1. The statistic reference of indicators. [file 12887_2022_3718_MOESM2_ESM.docx]

| **Table S1**. The statistic reference of indicators. | |
| --- | --- |
| Indicator | statistic reference |
| Anemia | |
| 7~59 months old | <110 g/L |
| 5~11 years old | <115 g/L |
| 12~14 years old | <120 g/L |
| Standards of microelements | |
| Cu | 11.8~39.3 μmol/L |
| Ca | 1.55~2.10 mmol/L |
| Mg | 1.12~2.06 mmol/L |
| Fe | 7.52~11.82 mmol/L |
| Zn |  |
| 0~0.99 years old | 58~100 μmol/L |
| 1~1.99 years old | 62~110 μmol/L |
| 2~2.99 years old | 66~120 μmol/L |
| 3~4.99 years old | 72~130 μmol/L |
| ≥5 years old | 76.5~150 μmol/L |
| Pb | 0~100 μg/L |
| Cd | 0~5 μg/L |
| Intoxication levels |  |
| Pb | >100 μg/L |
| Cd | >2.5 μg/L |
| Anti-HBs |  |
| Positive | >10mIU/mL |
| Negative | ≤10mIU/mL |
