# Supplementary material for: Evaluation of factors associated the expression of anti-HBs in children in Hunan Province, China
Source: BMC Pediatr. 2022 Dec 6;22:697. doi: 10.1186/s12887-022-03718-z (PMC9724425; doi:10.1186/s12887-022-03718-z)
Supplement: Supplementary file 3 — Additional file 3: Table S2. The statistic reference of BMI. [file 12887_2022_3718_MOESM3_ESM.docx]

**Table S2.** The statistic reference of BMI.

| Age | Normal BMI range | |
| --- | --- | --- |
|  | Male | Female |
| 6~6.99 years | 13.1-17 | 12.7~17.3 |
| 7~7.99 years | 13.3~17.4 | 12.9~17.7 |
| 8~8.99 years | 13.5-18 | 13.1~18.4 |
| 9~9.99 years | 13.7~18.5 | 13.5~19 |
| 10~10.99 years | 14~19.2 | 13.9~19.9 |
| 11~11.99 years | 14.5~20 | 14.5~20.8 |
| 12~12.99 years | 15~20.9 | 15~21.8 |
| 13~13.99 years | 15.5~21.7 | 15.5~22.7 |
| 14~14.99 years | 16~22.4 | 16.2~23 |
| 15~15.99 years | 16.8~23 | 17.1~23.4 |
| 16~16.99 years | 17.5~23.5 | 17.6~23.7 |
| 17~18 years | 18~24 | 18~24 |

The children whose BMI was lower than the reference range were considered as thin, while BMI was higher than reference range were considered as overweight.
